# Supplementary material for: The Temporal Change in Ionised Calcium, Parathyroid Hormone and Bone Metabolism Following Ingestion of a Plant-Sourced Marine Mineral + Protein Isolate in Healthy Young Adults
Source: Nutrients. 2024 Sep 14;16(18):3110. doi: 10.3390/nu16183110 (PMC11434972; doi:10.3390/nu16183110)
Supplement: Supplementary file 1 [file nutrients-16-03110-s001.zip › Supplementary Figure S1.pdf]

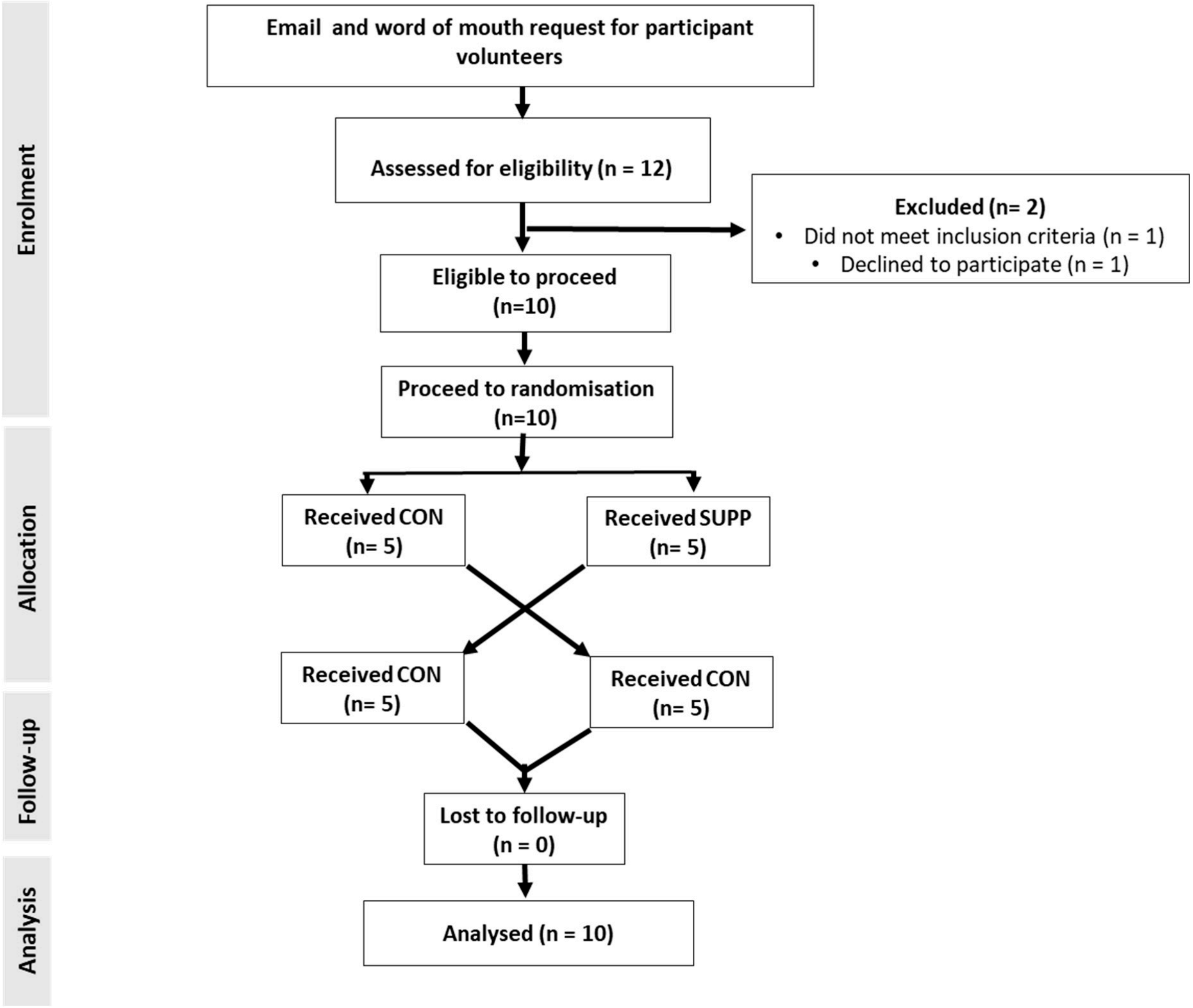

Supplementary Figure S1. CONSORT flow diagram of participant enrolment, allocation, follow-up, and analysis.
