# Supplementary material for: The Temporal Change in Ionised Calcium, Parathyroid Hormone and Bone Metabolism Following Ingestion of a Plant-Sourced Marine Mineral + Protein Isolate in Healthy Young Adults
Source: Nutrients. 2024 Sep 14;16(18):3110. doi: 10.3390/nu16183110 (PMC11434972; doi:10.3390/nu16183110)
Supplement: Supplementary file 1 [file nutrients-16-03110-s001.zip › Supplementary Table S1.pdf]

Supplementary Table S1. Compositional analysis of the plant-sourced mineral + protein supplement.

***Aquamin F Trace Mineral Analysis**			
**Analyte**	**mg/kg**	**Analyte**	**mg/kg**
Aluminium	150	Neodymium	0.5
Antimony	0.043	Nickel	0.95
Arsenic	0.56	Niobium	0.069
Barium	5.07	Osmium	< 0.004
Beryllium	0.068	Palladium	0.58
Bismuth	< 0.001	Phosphorus	440
Boron	37.8	Platinum	< 0.002
Bromine	< 20	Potassium	123
Cadmium	0.579	Praseodymium	0.107
Caesium	0.001	Rhenium	< 0.001
Calcium	320000	Rhodium	0.017
Cerium	0.822	Rubidium	0.03
Chlorine	< 10000	Ruthenium	0.004
Chromium	1.84	Samarium	0.114
Cobalt	0.174	Scandium	0.543
Copper	1.2	Selenium	0.07
Dysprosium	0.162	Silicon	400
Erbium	0.11	Silver	< 0.01
Europium	0.036	Sodium	4150
Gadolinium	0.153	Strontium	2110
Gallium	0.09	Sulphur	2960
Germanium	0.04	Tantalum	0.002
Gold	< 0.005	Tellurium	< 0.1
Hafnium	0.029	Terbium	0.024
Holmium	0.036	Thallium	0.006
Iodine	16.4	Thorium	0.007
Iridium	< 0.001	Thulium	0.016
Iron	604	Tin	0.02
Lanthanum	0.497	Titanium	50
Lead	0.19	Tungsten	0.014
Lithium	3.32	Vanadium	8.49
Lutetium	0.016	Ytterbium	0.131
Magnesium	25900	Yttrium	1.33
Manganese	37.2	Zinc	2
Mercury	< 0.005	Zirconium	2.16
Molybdenum	0.615		

***Method summary:** Aliquots of homogenised test sample were digested in a mixture of nitric acid and hydrochloric acid using a high-pressure microwave system. Quantification was by inductively coupled plasma-mass spectrometry (ICP-MS) with collision cell.

**Fava bean protein isolate**	
	**g/100g**
Moisture, 105°C	4%
Ash, 450°C	6.6%
Total energy (kcal/100g)	380
Fat	5.1
Carbohydrate (g/100g)	3.1
Protein (Dry basis)	78%
Amino acid	
Histidine	1.91
Isoleucine	3
Leucine	5.66
Lysine	4.81
Methionine + cysteine	1.29
Methionine	0.524
Cysteine	0.77
Phenylalanine + tyrosine	5.87
Phenylalanine	3.35
Tyrosine	2.52
Threonine	2.56
Tryptophan	0.61
Valine	3.37
Σ Indispensable	29
% Indispensable	42%
Alanine	2.96
Arginine	6.52
Aspartic acid	8.35
Glutamic acid	12.4
Glycine	2.94
Proline	3.24
Serine	3.76
Σ Dispensable (g)	40
% Dispensable	58%
Total AA (g)	69
